# Fatal cases associated with eating chapatti contaminated with organophosphate in Tororo District, Eastern Uganda, 2015: case series

**DOI:** 10.1186/s12889-019-7143-0

**Published:** 2019-06-17

**Authors:** Benon Kwesiga, Alex R. Ario, Lilian Bulage, Julie Harris, Bao-Ping Zhu

**Affiliations:** 1 Uganda Public Health Fellowship Program, Ministry of Health, P.O. Box 7272, Kampala, Uganda; 2 United States Centres for Disease Control and Prevention, Kampala, Uganda; 3 Division of Global Health Protection, Center for Global Health, United States Centers for Disease Control and Prevention, Atlanta, GA USA

**Keywords:** Organophosphate, Poisoning, Pesticide, Uganda

## Abstract

**Background:**

Few cases of organophosphate poisoning in developing countries have been investigated using clinical and epidemiological methods. On 30 October 2015, 3 students at Mukuju School, Tororo District, Uganda, died soon after eating chapatti (locally-made flat bread) from the same food stand. Ministry of Health investigated to identify the cause and recommend prevention measures.

**Methods:**

We defined a case as onset during 30–31 October 2015 in a resident of Mukuju Town of ≥1 of the following symptoms: excessive saliva, profuse sweating, dizziness, low blood pressure, constricted pupils or loss of consciousness. We reviewed medical/police records and interviewed survivors, healthcare workers, and police officers. We collected samples of implicated food for toxicological analysis. Autopsies were performed on decedents to identify the cause of death.

**Results:**

We identified 7 cases with 3 deaths (case-fatality ratio = 43%). Clinical manifestations included acute onset of confusion (100%), constricted pupils (43%), excessive saliva (43%), and low blood pressure (43%). All 7 cases had onset from 16:00–18:00 h on 30 October, with a point-source exposure pattern. Of the 7 cases, 86% (6/7) were men; the mean age was 24 (range: 20–32) years. The 3 decedents each ate a whole chapatti while the other 4 cases ate half or less. Autopsy findings of the 3 decedents indicated organophosphate poisoning. Toxicological analysis found high levels of malathion in leftover foods (266 mg/L in dough and 258 mg/L in chapatti) and malaoxon (a highly toxic malathion derivative) in decedents’ postmortem specimens (mean levels of 19 mg/L in the blood and 22 mg/L in the gastric contents). There was a delay of 4 h before the patients received appropriate treatment. Police investigations revealed that flour used to make the chapatti was intentionally contaminated with an organophosphate pesticide.

**Conclusion:**

This fatal outbreak of organophosphate poisoning was associated with consumption of roadside-vended chapatti made of flour contaminated with pesticide. Clinicians should be aware of symptoms of organophosphate poisoning and prepared to treat it quickly. Street vendors should carefully consider the source of their ingredients. An in-depth surveillance review of such poisonings in Uganda would guide policymakers in reducing access by criminals and accidental exposures for the public.

**Electronic supplementary material:**

The online version of this article (10.1186/s12889-019-7143-0) contains supplementary material, which is available to authorized users.

## Background

Organophosphate poisoning results from exposure to organophosphates which cause the inhibition of acetylcholinesterase, a critical compound for nerve function [1–3]. The irreversible blockage of this enzyme causes acetylcholine accumulation in the body that can result in muscle overstimulation [3] and acute symptoms such as muscle weakness, fatigue, muscle cramps, fasciculation, vomiting, diarrhoea and paralysis [2]. Beyond this, people suffering from organophosphate poisoning can experience anxiety, headache, convulsions, ataxia, depression of respiration and circulation, tremor, general weakness, tightness in the chest, wheezing due to bronchoconstriction, increased bronchial secretions, increased salivation, lacrimation, sweating, peristalsis, urination, and potentially coma [2]. The onset and severity of symptoms, whether acute or chronic, depends upon the specific organophosphate, the route of exposure, the dose, and the individual’s ability to degrade the compound [4]. Food contaminated by organophosphates might have a chemical smell and bitter taste although this depends on the amount of organophosphate.

Organophosphates are one of the commonest agents of poisoning worldwide: there are an estimated one million organophosphate poisonings per year worldwide, with several hundred thousand resulting in fatalities [5]. Organophosphates are frequently used in suicides and homicides [5]. Unintentional organophosphate poisoning most commonly occurs among agricultural workers or children [6–8]. Poisoning has also been caused by ingestion of contaminated flour as well as leafy vegetables on which the organophosphates were used [9, 10]. In the treatment of organophosphate poisoning, timely decontamination is key. Pralidoxime and anticholinergic drugs such as atropine counteract the effects of excess acetylcholine and reactivate acetylcholinesterase [5, 11]. Another treatment involves management of complications such as aspiration, coma, etc. as they arise [12]. Organophosphates can survive for long periods in the environment and can withstand high temperatures during cooking [13].

In Uganda, organophosphate poisoning is a common occurrence [14]. These chemicals are sold by animal drug shops, and shops that sell general merchandise. Pesticides are used in most homes, for indoor spraying, and on agricultural farms. In Uganda, organophosphate pesticides are available in both powder and liquid forms. A recent study reviewing pesticide poisoning cases treated in health facilities in Uganda found that organophosphate pesticides accounted for 73% of the poisonings [15]. This study also found that the case-fatality rate was higher in rural health facilities than in urban facilities because the urban health facilities provide better intensive care. It recommended restriction of pesticide availability, an intervention that has proven to be effective in low-income countries.

On 30 October 2015, 3 students at Mukuju Primary Teachers’ College in Tororo District, Eastern Uganda, died, reportedly after eating chapatti (locally-made flat bread) from a roadside chapatti vendor near their school. Chapatti is flattened bread made by frying a paste made from wheat flour and water in oil. Within minutes of eating the chapatti, the students developed severe symptoms; all three died while being treated in a nearby health centre. In this case report, we used clinical and epidemiological investigations to ascertain the cause of death and inform public health actions to prevent future incidents. There are a few cases of organophosphate poisoning in developing countries that have been comprehensively investigated using both clinical and epidemiological methods [16–18].

## Methods

We defined a suspected case as sudden onset of foaming at the mouth of saliva, low blood pressure, loss of consciousness, or constricted pupils in a resident of Mukuju village from 24 October 2015 onwards. We developed a questionnaire to guide the interviewers during data collection and it is attached as Additional file 1. We searched for additional cases at nearby health centres and district hospitals, at the school with the help of the school administrators, and, with the help of a village chief, among persons living near the chapatti vending area. We reviewed patient records at the hospital and postmortem results obtained from the police to verify the clinical characteristics of patients. Data collected for this investigation was not publicly available. However, being the Uganda ministry of health investigation team that was authorised to conduct this investigation, we were allowed access to this data. We were therefore granted access to all hospital and police records that were relevant to this emergency public health investigation.

We assessed whether a dose-response relationship existed by relating the number of symptoms each case-patient developed with the amount of chapatti eaten. On average, a chapatti made in Uganda has a diameter of 10 cm and weighs approximately 100 g.

The police surgeon undertook postmortem examination to determine the cause of deaths of the decedents and collected the decedents’ blood and gastric contents for toxicological testing. With the help of the police, we also conducted an environmental assessment of the chapatti vending business to assess for possible sources of contamination, and collected leftover baking flour, cooking oil, chapatti and flour-dough specimens for toxicological examination. Toxicological testing was conducted at the Government Analytical Laboratory in Kampala (Uganda’s capital city); the laboratory used liquid chromatography triple quadrupole mass spectrometry [19] to determine the level of organophosphates in the specimens. Police conducted criminal investigations by interviewing identified suspects and survivors.

## Results

**Fig. 1:**
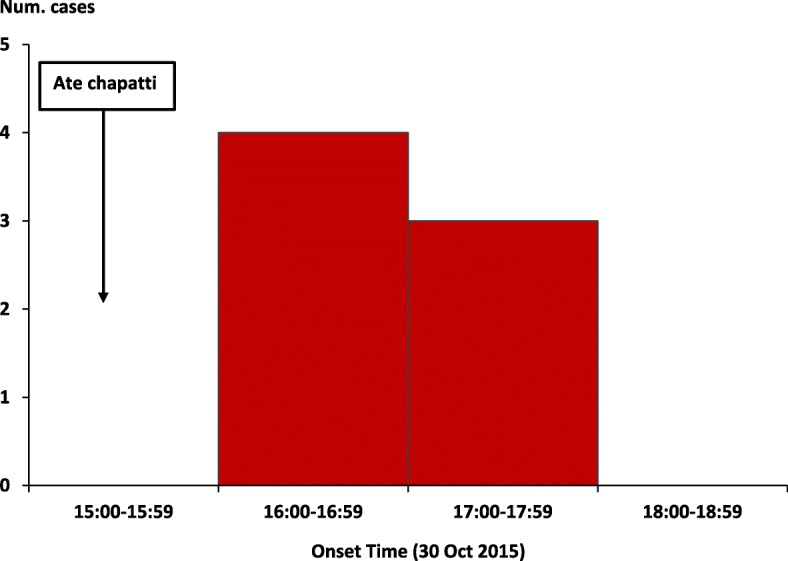
Symptom onset of 7 cases of organophosphate poisoning: Mukuju Village, Tororo District, Uganda, 30 October 2015

**Fig. 2:**
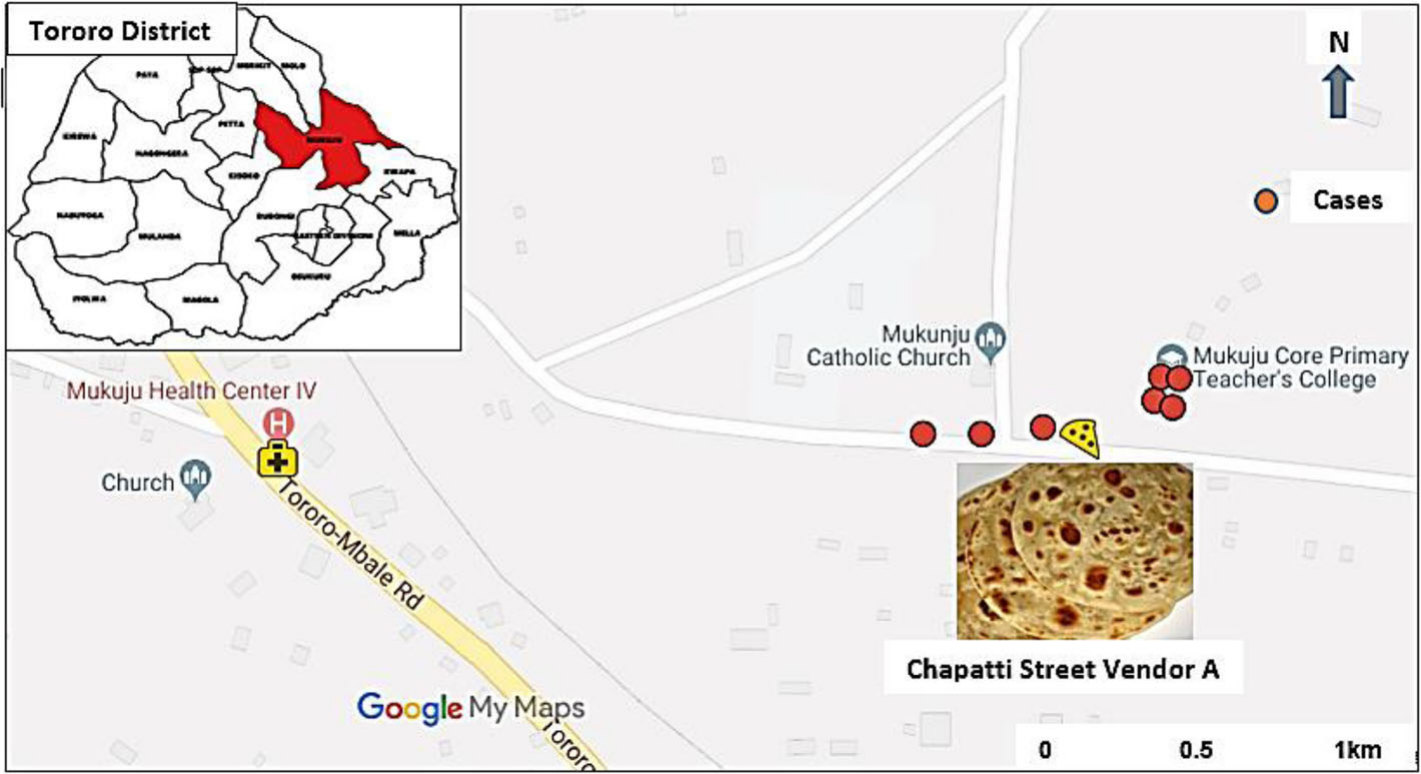
Map showing place of residence of cases of organophosphate poisoning: Mukuju Village, Tororo District, Uganda, 3[20]0 October 2015. This map was drawn using Google maps, an online open-source software used for mapping purposes [20]

We identified 7 suspected cases, with the onset of symptoms occurring between 16:00 h and 18:00 h on 30 October 2015 (Fig. 1). Three of the 7 case-patients died (case-fatality rate = 43%). Of the 7 suspected case-patients, six (86%) were men. The mean age was 24 (range: 20–32) years, and four (57%) case-patients (including all 3 deceased) were students. The other three case-patients were a chapatti vendor, a Boda-Boda (i.e., motorcycle taxi) driver, and a hair salon worker. All case-patients were from the same village, located near the roadside chapatti vending place which was just outside the school gate (Figs. 2, [20]. Students and other community members in the area often bought chapatti from this vendor.

**Fig. 3:**
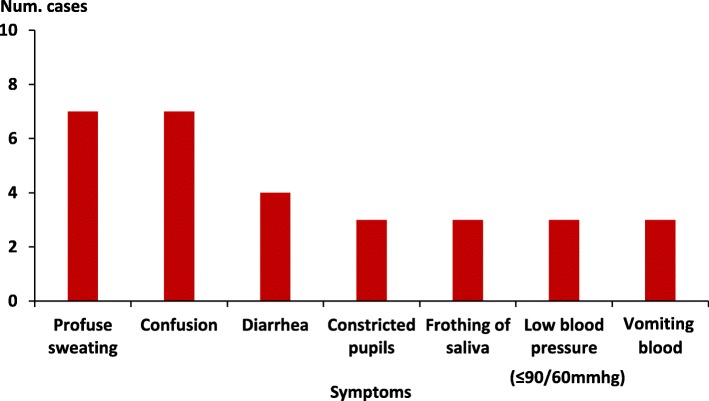
Distribution of symptoms among 7 cases of organophosphate poisoning: Mukuju Village, Tororo District, Uganda, 30 October 2015

This community had several chapatti vendors located at strategic trading points within its villages. Mukuju village, where the outbreak occurred, had only one chapatti vendor (Vendor A). Vendor A made and sold chapatti in Mukuju village every day. He would sell approximately 50 chapatti every day. During the morning of 30 October 2015, chapatti were sold from the vending place in the normal fashion, with no reported problems. However, in the afternoon, 4 students each bought a chapatti from Vendor A. Three ate the whole chapatti, while one ate only part of it because it ‘tasted strange’; this student returned the chapatti to Vendor A. Upon hearing the student’s complaint, Vendor A tasted the chapatti. All four students and Vendor A developed symptoms approximately 20 min after eating the chapatti. The initial symptoms included profuse sweating, foaming of saliva, confusion, vomiting, diarrhoea, and difficulty breathing, followed by more severe symptoms and signs; low blood pressure (< 90/60 mmHg), altered state of consciousness, and constricted pupils (Fig. 3). Severity increased with the amount of chapatti eaten (Table 1).

**Table 1:** Clinical presentation and outcome of 7 case-patients in relation to the amount of chapatti eaten during an outbreak of organophosphate poisoning: Mukuju Village, Tororo District, Uganda, 30 October 2015

Case No.	Age	Sex	Occupation	Amount of chapatti eaten	Symptoms and signs	Outcome
1	23	M	Student	1	Profuse sweating, foaming of saliva, vomiting blood, diarrhoea, constricted pupils, low blood pressure and coma	Died
2	20	M	Student	1	Profuse sweating, foaming of saliva, vomiting blood, diarrhoea, constricted pupils, low blood pressure and coma	Died
3	20	M	Student	1	Profuse sweating, foaming of saliva, vomiting blood, diarrhoea, constricted pupils, low blood pressure and coma	Died
4	26	M	Student	Part of a chapatti	Dizziness, Profuse sweating, confusion,	Recovered
5	24	M	Chapati Vendor	Part of a chapatti	Dizziness, profuse sweating	Recovered
6	25	M	Boda-Boda driver	Part of a chapatti	Dizziness, profuse sweating, diarrhoea	Recovered
7	32	F	Hair-salon worker	A little flour	Dizziness, profuse sweating	Recovered

The students were rushed to nearby Mukuju Health Centre IV where they were initially administered intravenous fluids while health workers tried to identify the cause of illness. After approximately 4 h at this health centre with no clear diagnosis, three of the four students lost consciousness and were referred to the nearby Tororo Hospital. The clinician at Tororo Hospital who treated the students noticed a strange smell while examining the patients and suspected pesticide poisoning. The 3 students who had eaten a whole chapatti were all treated with atropine and pralidoxime to reverse the cholinergic effects of organophosphate poisoning on the body, but they died shortly after admission (Table 1). The student who had eaten only a portion of his chapatti recovered fully and left the hospital after 2 days. The other three case-patients, including Vendor A, a Boda-Boda (“motorcycle taxi”) rider, and a hair-salon worker, each of whom ate part of a chapatti from Vendor A, made a full recovery (Table 1).

Autopsy records of the 3 decedents indicated that the gut contents had a strong organophosphate smell and evidence of multiple organ failure. The toxicological analysis found high levels of malathion in the leftover food specimens (266 mg/L in the dough and 258 mg/L in the chapatti). The United States Environmental Protection Agency has established a malathion acute reference dose of 0.14 mg/kg/day [21]. We also found high levels of malaoxon, a highly toxic metabolic derivative of malathion, in the 3 decedents’ postmortem specimens (average of 19 mg/L in the blood and 22 mg/L in the gastric contents) [22, 23].

Police investigations revealed that Vendor A occasionally accepted flour from his clients to make chapatti for them. On the day of the poisoning, Vendor A received approximately 1 kg of flour from one of his clients, which he then used to make the implicated chapatti. Police investigations identified the person who provided the contaminated flour to the vendor and concluded that the contamination was intentional. The individual was arrested and convicted of murder, and sentenced to prison for 14 years. Unfortunately, we were unable to obtain details of how the contamination actually occurred.

## Discussion

This outbreak of organophosphate poisoning was caused by eating street-vended chapatti made from flour contaminated with pesticide powder. The symptoms of the patients, as well as findings of epidemiologic, laboratory, and environmental investigations were all consistent with malathion organophosphate poisoning. This outbreak investigation and response demonstrated that good collaboration between police and public health agencies during criminal outbreak investigations can benefit both sides and such collaboration reflects a core component of global health security.

Malathion is a common, broad-spectrum organophosphate pesticide used to control a variety of outdoor pests in both agricultural and residential settings [24]. It is readily available in Uganda and other developing countries as a pesticide for use in homes and agricultural farms. While malathion itself has low toxicity among humans, its metabolite malaoxon can be up to 20 times more toxic [23]. Malaoxon toxicity in humans is not a well-studied area. During a study on malathion and malaoxon toxicity in humans, autopsy samples from an individual who had ingested large quantities of malathion were analysed for malaoxon [25]. Malaoxon was identified at low levels in some tissues but the highest concentration was in fat (8.2 mg/l) [25]. Compared to our investigation, these malaoxon levels were less than half of the concentrations we found in blood and gastric contents of the 3 descents indicating a high level of toxicity in these cases. Although organophosphates are rarely associated with human outbreaks, a few documented outbreaks exist, including one in India (60 men ate chapatti containing malathion and 1 person died [3]) and another in two farming villages in Bangladesh, which resulted in 2 deaths [16]. Incidents of acute unintentional organophosphate poisoning (due to monocrotophos) after consumption of contaminated millet flour and oil have also been documented in India [26, 27]. There are few reports about organophosphate poisoning with criminal intent [28].

In this outbreak, the 4-h delay in receipt of appropriate treatment likely contributed to the high case-fatality rate among case-patients. Had health workers quickly confirmed the diagnosis and provided specific treatment or referred the patients to higher-level health facility sooner, the 3 students might have had a better outcome [17]. Public and clinical education about warning signs of organophosphate poisoning might have facilitated faster recognition of the syndrome. The easy availability of organophosphate compounds in Uganda could make this type of education particularly important in this setting.

Consumption of street-vended food is common in Uganda and in most developing countries. Street-vended food is often considered risky due to lack of regulation and the likelihood of contamination with foodborne bacteria and viruses. Although rare, our investigation revealed additional risks of eating food for which the ingredients have unknown provenance, including street food. Although police investigations concluded that the flour poisoning was intentional in this outbreak, we were unfortunately unable to obtain details of how the accused contaminated the flour. However, additional education to street food vendors that emphasizes only making food with ingredients that the vendor has purchased himself or herself may be warranted.

Despite the fact that greater volumes of pesticides are used in developed than in developing countries, developing countries suffer pesticide poisoning much more frequently, largely due to the lack of training on their use and the absence of regulatory laws [29]. Uganda is one of several African countries without a policy to regulate access to pesticides. While it is inappropriate to make specific public health recommendations in response to an atypical intentional event such as the one described in this paper, a more in-depth surveillance review of such poisonings in Uganda could help generate evidence of the linkage between intentional pesticide poisonings and access. Such evidence could guide policymakers to reduce pesticide access by criminals and accidental exposures for the public.

We conducted the investigation a few days after the incident. By the time of the investigation, most of the patients had already recovered and discharged, and clinical specimens were not taken for testing while they were hospitalized. We were, therefore, unable to test blood acetylcholinesterase levels for any of the patients, which could have confirmed organophosphate poisoning in their blood. Despite this, the clinical and epidemiological evidence, autopsy findings and testing of environmental samples provided sufficient evidence of organophosphate poisoning.

## Conclusion

This fatal outbreak of organophosphate poisoning was associated with consumption of roadside-vended food (chapatti) made of flour contaminated with pesticide. Following the investigation, all chapatti-vending points within the village were closed for inspection. The Tororo District Health Team retrained health workers on early diagnosis and referral to prevent delays in such patients. The District health Team and Police cautioned roadside food vendors about accepting flour from clients and the dangers of organophosphate poisoning and advised them to only make food with ingredients they have purchased themselves. We recommend an in-depth surveillance review of such poisonings in Uganda to help guide policymakers to reduce access by criminals and accidental exposures for the public**.**

## Additional file

Additional file 1: Questionnaire guide for interviewer. Demographic characteristics, clinical information and food history of patients during a fatal food poisoning incident in Tororo District, Uganda, 2015. This tool was used to collect data for Table 1, and Figs. 1, 2 and 3. The questionnaire that was used to guide interviewers while collecting data about the patients’ demographic characteristics, clinical presentation and food intake history on the day of the food poisoning incident (30 October 2015). (DOCX 18 kb)

## Data Availability

All data generated and analyzed from this investigation are available upon reasonable request from the corresponding author.
