# Supplementary material for: Fatal cases associated with eating chapatti contaminated with organophosphate in Tororo District, Eastern Uganda, 2015: case series
Source: BMC Public Health. 2019 Jun 17;19:767. doi: 10.1186/s12889-019-7143-0 (PMC6580625; doi:10.1186/s12889-019-7143-0)
Supplement: Supplementary file 1 — Questionnaire guide for interviewer. Demographic characteristics, clinical information and food history of patients during a fatal food poisoning incident in Tororo District, Uganda, 2015. This tool was used to collect data for Table 1, and Figs. 1, 2 and 3. The questionnaire that was used to guide interviewers while collecting data about the patients’ demographic characteristics, clinical presentation and food intake history on the day of the food poisoning incident (30 October 2015). (DOCX 18 kb) [file 12889_2019_7143_MOESM1_ESM.docx]

**Questionnaire guide for interviewer**

**Investigation of fatal food poisoning in Mukuju village, Tororo District, Uganda**

Interviewer’s name: ………………………………

Date:………………………………………Time:………………………………

Case I.D: …………………………………

**Brief introduction:**

On 30 October 2015, 3 students in Mukuju Primary Teacher’s College, Tororo district, died of what appeared to be food poisoning. All 3 are reported to have eaten chapatti from the same food-stand for lunch before falling sick. Tororo hospital where these patients died, notified Tororo District Health Officer who in turn notified Ministry of Health and asked them to help with the investigation. You have been identified as one of the people who was affected by this incident and I would like to ask you a few questions about your unfortunate experience. The information you provide will help us find the cause of this incident and prevent similar incidents in future.

**Demographic data**

Names: …………………………… Telephone contact: ……………………………….

Age: ………………….. Gender: ………………… Occupation: ……………………

Coordinates of patient’s household: …………………….. Village: ………………………

Parish: ………………………. Sub county: ………………… District: ……………….

**Clinical presentation**

1. When did the patient notice the first symptom? Date: ………….. Time: …………….
2. Did the patient experience any of the following signs and symptoms? *(You will need to obtain some of this information from hospital staff and clinical records. Tick all that apply)*

Fever 🞎 Headache 🞎 Dizziness 🞎 Confusion 🞎

Profuse sweating 🞎 Vomiting 🞎 Vomiting blood 🞎 Diarrhea 🞎

Foaming of saliva 🞎 Constricted pupils 🞎 Low blood pressure 🞎

Loss of consciousness 🞎 Others 🞎 (Specify……………….)

1. Was the patient hospitalized? 🞎

If yes to 3 above, which health facility? *(You will need to obtain some of this information from hospital staff and clinical records)*

-How long was the patient hospitalized? ……………………………

-What treatment was given to the patient? …………………………….

-Were laboratory tests done? ……… (If yes, state which ones and their results)

……………………………………………………………………………………….

1. What is the patient’s status: Alive: 🞎 Dead: 🞎
2. If patient died, was postmortem done? What were the results of the postmortem? *(Inquire from hospital and police)*

**Food history on 30 October 2015**

1. What foods or drinks did the patient eat on the Friday of 30 October 2015?

| **Food or drink item** | **Yes/No** | **Approximate time food or drink was eaten or drunk (xx:xx hours)** |
| --- | --- | --- |
| Chapati from Vendor X |  |  |
| Chapati from elsewhere |  |  |
| Other foods or drinks (Specify) |  |  |
| 1. …………….. |  |  |
| 1. ……………. |  |  |
| 1. …………… |  |  |

1. If the patient ate chapati from vendor X on 30 October 2015, do they still have any leftover of the chapatti? *(Ask if you can collect it for testing)*
